# Sigmoid Sinus Thrombosis Associated to Chronic Otitis Media

**DOI:** 10.1016/S1808-8694(15)31062-4

**Published:** 2015-10-22

**Authors:** Norma de Oliveira Penido, Ronaldo Nunes Toledo, Paula Angélica Lorenzon Silveira, Mario Sérgio Lei Munhoz, José Ricardo Gurgel Testa, Oswaldo Laércio Mendonça Cruz

**Affiliations:** 1 PhD in Medicine. Associate Professor Unifesp-Epm.; 2 M.S. in Otorhinolaryngology - UNIFESP, PhD student in Sciences - UNIFESP.; 3 Otorhinolaryngologist. M.S. student - UNIFESP-EPM.; 4 Associate Professor of Otorhinolaryngology - Federal University of São Paulo - Paulista School of Medicine. UNIFESP-EPM.; 5 PhD in Medicine. Professor of Otorhinolaryngology - Federal University of São Paulo - Paulista School of Medicine. UNIFESP-EPM.; 6 Associate Professor of Otorhinolaryngology - Federal University of São Paulo - Paulista School of Medicine. UNIFESP-EPM.; 7 Department of Otorhinolaryngology and Head and Neck Surgery - Federal University of São Paulo - Paulista School of Medicine, Mailing Address: Norma de Oliveira Penido - Rua René Zanlutti 160 apt. 131 Chácara Klabin 04116-260 São Paulo SP; 8 Department of Otorhinolaryngology and Head and Neck Surgery - Federal University of São Paulo - Paulista School of Medicine.

**Keywords:** complications, mastoiditis, otitis media, intracranial sinus thrombosis

## Summary

**O**togenous lateral sinus thrombosis (OLST) is a rare disease and presents a controversial treatment. **Aim:** Clinical aspects and treatment were reported based on our experience. **Methods:** Retrospective study. Six cases of OLST were treated in our institution in the last ten years. Clinical and imaging data were analyzed. **Results:** All six patients had the lateral sinus thrombosis detected during image evaluation for other symptoms related to chronic otitis media (COM) complications. Fever, headache and facial paralysis were the main clinical manifestation related to mastoiditis, meningitis and cerebellar abscess. We could not identify, in any case, specific features of lateral sinus thrombosis. In all cases a mastoidectomy was associated with large spectrum antibiotics maintained for 3 months. In three cases anticoagulation therapy was introduced and in three cases anticoagulation was not indicated. All cases presented a good clinical evolution, without sequelae. **Conclusions:** OLST is almost always associated with other complications of COM. It is diagnosed almost by accident during the investigative image study. We believe such disease is underestimated. In our experience, OLST presents a benign course, and mastoidectomy with antibiotics is the treatment of choice.

## INTRODUCTION

Sigmoid sinus thrombosis is a rare entity, usually secondary to thrombophilia, head injuries, neoplasias, use of injectable drugs and, mainly, infectious diseases.^1^,^2^ When the etiology is infectious, it is called sigmoid sinus septic thrombosis (SSST), and middle ear chronic or acute diseases represent the most frequent causes. After antibiotic introduction and improvement, not only its incidence has dropped dramatically, but also its mortality rate.^3^

Large spectrum antibiotics that penetrate the blood-brain barrier are used for extended time in the treatment of SSST. In those cases secondary to chronic otitis media, it is fundamental to surgically approach the infection, usually through a mastoidectomy. However, the thrombus approach is still controversial, according to the literature. There are authors who advocate sinus opening with thrombectomy and jugular vein ligation,^1^,^4^,^5^,^6^ and there are others who advocate only the surgical clearance around the sigmoid sinus, together with infection surgical drainage.^7^ Even more controversial is the use of anticoagulants such as heparin, or oral anticoagulant agents. The latter has been seen as a therapy recommended in order to prevent the complications related to the thrombus persistence or its spread; others say there are few studies carried out in cases of sigmoid sinus otogenic thrombosis that could advocate its benefits. ^1^,^5^, ^6^, ^7^, ^8^, ^9^, ^10^ As to a possible embolization, venous infarctions and septic thrombophlebitis have shown a marked decrease in its incidence with proper antibiotic coverage only, not justifying anticoagulation risks, especially: bleeding, drug interactions, thrombocytopenia, osteoporosis and skin hemorrhagic necrosis.^11^.

In the present paper we report on our experience in the diagnosis, treatment and follow up of SSST cases seen during a ten year period in our institution.

## METHODS

We carried out a retrospective analysis reporting our experience in treating SSST cases between the years of 1993 and 2003. All patients with diagnostic proof of SSST by MR angiography or arteriography; and a long follow up, ranging from 6 months to 6 years.

## RESULTS

Between 1993 and 2003, we diagnosed and treated 6 cases of SSST in our institution (Charts 1 and 2). Most were young patients, with mean age of 26 years, and extreme ages of 10 and 50 years. The left side was the one more frequently affected in five patients, and only one patient had his right side affected. Males (4) prevailed over females (2).

**Chart 1:** Diagnosis and complementary tests.

PACIENT	AGE AND GENDER	SIDE	ETIOLOGY	DIAGNOSTIC	EXAM	RELATED COMPLICATIONS
1-JCS	24 M	Left	OMCC	Postop	CT; MR angiography	Mastoiditis Brain abscess Meningitis
2-MSA	25 F	Left	OMCNC	Postop	CT Angiography	Mastoiditis Brain abscess VI cranial nerve palsy
3-DGPV	17 M	Right	OMCC	Preop	CT; MR angiography	Mastoiditis Brain abscess cerebellar Meningitis
4-FGS	50 M	Left	OMCC	Preop	CT; MR angiography	Mastoiditis Meningitis
5-APS	30 F	Left	OMCC	Preop	CT; MR angiography	Mastoiditis Subdural abscess
6-RO	10 M	Left	OMCC	Intraoperative	CT; MR angiography	Mastoiditis Facial paralysis

M - Male CCOM - Cholesteatomatous Otitis Media

F - Female NCCOM- Non-Cholesteatomatous Otitis Media

CT - Computerized Tomography

**Chart 2:** Treatment and Evolution

PACIENT	CULTURE	SURGERY	ANTIBIOTIC	ANTICOAGULANT	EVOLUTION
1-JCS	Enterococcus sp	Mastoidectomy Canal wall down	Ceftriaxone Metronidazole Vancomycin Gentamicin	Heparin (30) days Oral anticoagulant Six months	Good clinical outcome No sequelae
2-MSA	Negative	Mastoidectomy Canal wall up Needle aspiration	Ceftriaxone Metronidazole	Heparin (28) days Oral anticoagulant Four months	Good outcome MR angiography without flow after 6 months
3-DGPS	Pseudomonas Aeruginosa	Mastoidectomy Canal wall down craniotomy Needle aspiration	Ceftriaxone Metronidazole Vancomycin	Heparin (30) days	Good outcome MR angiography without flow after 8 months
4-FGS	Proteus Mirabilis	Mastoidectomy Canal wall down Needle aspiration	Ceftriaxone Metronidazole	None	Good outcome MR angiography without flow after 4 years
5- APS	Negative	Mastoidectomy Canal wall down Needle aspiration	Ceftriaxone Clindamycin	None	Good outcome MR angiography without flow after 9 months
6-RO	Negative	Mastoidectomy Canal wall down Needle aspiration	Ceftriaxone Clindamycin	None	Good outcome MR angiography without flow after 6 months

All the patients had chronic middle ear disease, five of them had chronic cholesteatomatous otitis media (CCOM) and one of them had chronic suppurative otitis media (SCOM). All the patients had other alterations present besides SSST. All six patients had acute mastoiditis at the time of hospital admittance, and four of them also had intracranial abscesses, three had meningitis, one had peripheral facial paralysis and another had abducent nerve paralysis. The patients presented clinical symptoms more related to the other complications, especially those with meningitis and cerebral abscess, such as headache, stiff neck, high fever and malaise. We did not identify specific symptoms or any that would specifically suggest a SSST diagnosis.

**Figure 1:**
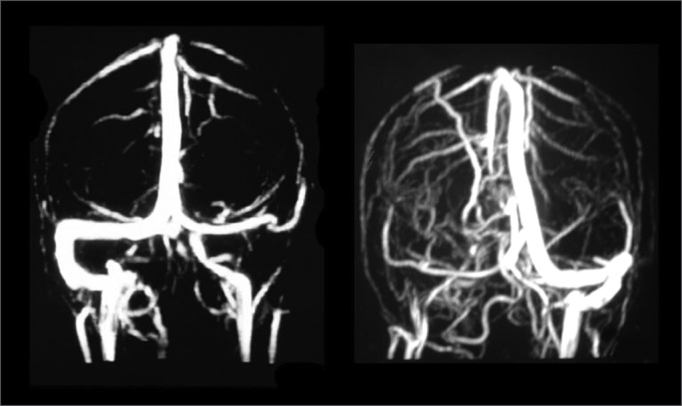
MR angiography showing two different cases of sigmoid sinus thrombosis. The left figure shows no flow in the right sigmoid sinus and the right side figure shows no flow in the left sinus, confirming the diagnosis of thrombosis.

Middle ear secretion culture was positive in 3 cases with growth of Enterococcus sp, Pseudomonas Aeruginosa and Proteus Mirabilis and negative in the other three; we believe we had such result because the patients used antibiotics. Patients were treated with broad spectrum antibiotics such as ceftriaxone associated with clindamycin and they were all surgically approached with canal wall down mastoidectomy for the cases of CCOM and canal wall up procedure for CSOM. During surgery we also cleared around the sigmoid sinus and removed granulation tissue and bone debris; however, without opening the sinus and directly approaching the thrombus. When SSST diagnosis was already known, it also again confirmed during surgery by means of a fine needle aspiration of the sigmoid sinus. The first three cases were treated with heparin and the three last cases without heparin or oral anticoagulation. Although all the patients underwent head and temporal bone CT Scans upon arrival at our service, SSST diagnostic suspicion only happened in the preoperative in half of the patients, the other half was diagnosed during surgery or in the postoperative. For SSST diagnostic confirmation we used MR angiography in five cases (Figure 1) and arteriography in one case.

All had favorable clinical outcome, with improvement in infection and surgical control of the ear disease, and there was only one case of CCOM recurrence about four years afterwards that needed another surgical approach. In five patients we carried out MR angiography studies later on and only one patient had recanalization of the sigmoid sinus. It is curious that this patient did not undergo treatment with heparin or anticoagulants. We had an extremely satisfactory clinical evolution of all six patients, regardless of sigmoid sinus recanalization.

## DISCUSSION

SSST is a severe complication of middle ear infectious diseases. In the past it bore high mortality rates, which was initially reduced with better surgical treatments, but mainly because of antibiotic therapy.^3^

It is believed that middle ear infection extends directly or through the mastoid emissary vein to the sigmoid sinus, resulting initially in perivascular micro abscess and the persistence of the infectious process; the micro abscesses end up involving the venous system, causing an infected thrombus.^11^

In our series, clinical symptoms were more associated with the other complications, such as meningitis and intracranial abscess, or to the ear problem such as mastoiditis and otorrhea. There doesn't seem to be specific clinical symptoms for SSST,1 however, in more extensive cases there usually is conscience level alteration and papilledema. Sometimes, it may progress forwardly to the internal jugular vein or backwardly to the transverse sinus, or to the superior or inferior petrous sinuses. When it hits the cavernous sinus, usually through the inferior petrous sinus, we may have abducent nerve palsy.10 In our cases, the diagnosis of SSST was always associated with other complications, and therefore, it is likely that we are underdiagnosing the isolated, less extensive cases.

**Figure 2:**
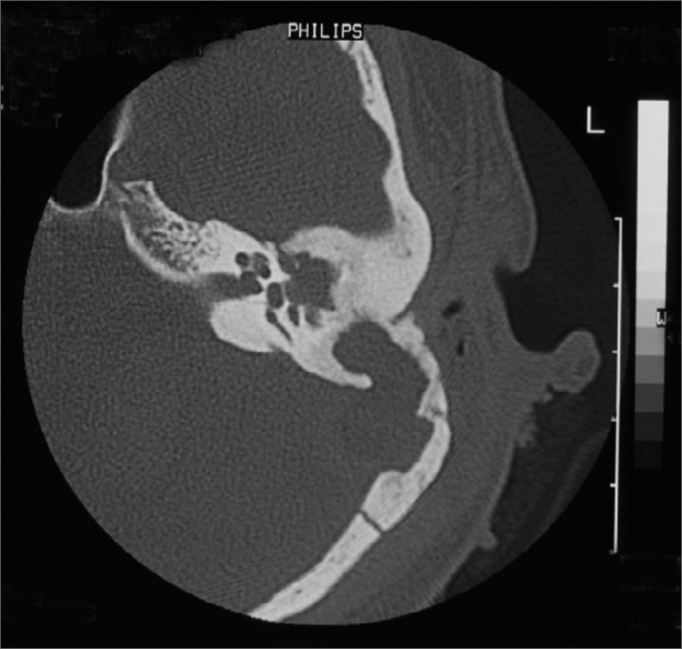
Temporal bones CT scan without contrast, showing extensive bone destruction in the sigmoid sinus area (dura mater of the posterior fossa).

**Figure 3:**
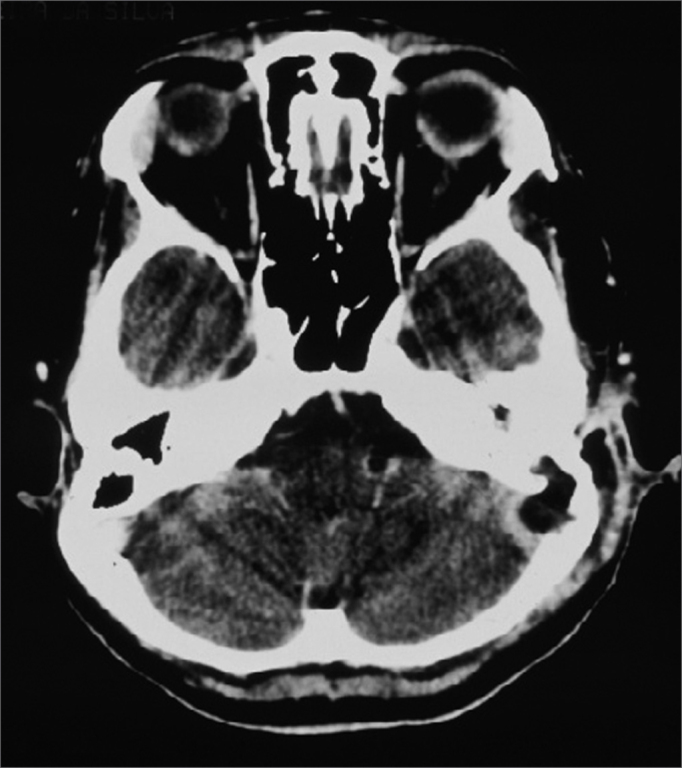
Skull CT scan with contrast, showing contrast highlight around the sigmoid sinus and lack of this highlight inside, with bone erosion to the posterior fossa.

**Figure 4:**
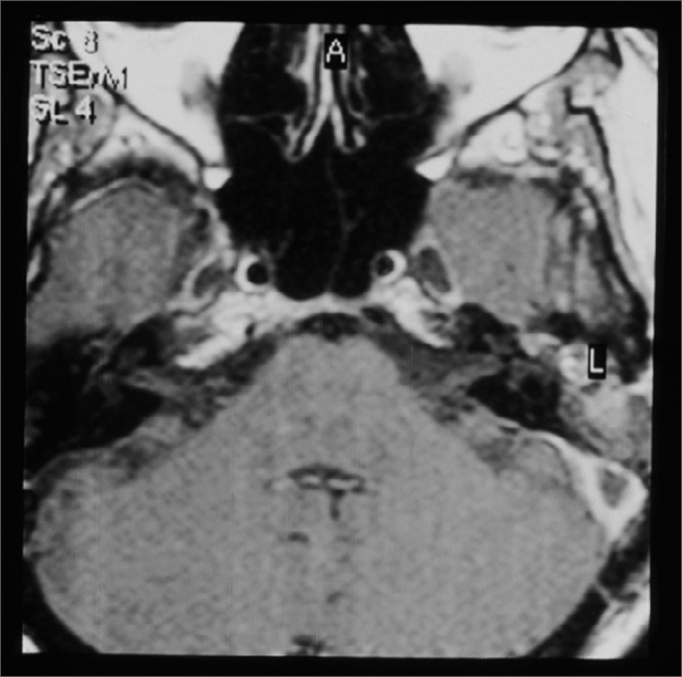
Temporal bone contrasted MRI, showing contrast highlight around the sigmoid sinus and no highlight inside, suggesting that there is a thrombus inside.

CT scan without contrast is the most used image study by otorhinolaryngologists in the assessment of patients with chronic otitis media, since it is an excellent exam to see bone changes; however, its sensitiveness is poor to assess cases of thrombosis. This was the first exam carried out in our patients, since they all had middle ear chronic disease and started with mastoiditis. Even then, in 3 cases, the SSST likelihood started to be appreciated after this exam (Figure 2), because of evidences of extensive bone destruction in the posterior fossa dura mater region, directly affecting the sigmoid sinus. In cases of otitis media complications, one should always order a CT scan with contrast (Figure 3), because if we see an image called “empty triangle” in the sigmoid sinus area, surrounded by the dura contrast- also called the “delta sign”- it is considered a typical sign of thrombosis.^5^ Nonetheless, this sign is not always detected and not all cases of thrombosis are documented with a CT scan. MRI is more sensitive to detect thrombosis, and better to assess blood flow, it may even show sinus obstruction outlining the lesion contours and the involvement of adjacent structures (Figure 4); however, for diagnostic confirmation we need MR angiography or arteriography. Classically, the arteriography is considered the exam with the best sensitivity and specificity for SSST diagnosis, however it does bear some restrictions because it is invasive and there is always the risk of contrast allergy. The MR angiography exam is much more sensitive than the CT scan^5^,^6^,^10^ and less invasive then arteriography in SSST diagnosis, that is why it is our favorite exam and it is currently used as a routine in our institutions in cases we suspect of SSST.

All our patients were treated by mastoidectomy, and when SSST was suspected in the postoperative, the sigmoid sinus was punctioned with a fine needle during surgery in order to confirm the diagnosis. We do not do the direct thrombus approach in any patient. There are authors who advocate aggressive surgical treatment such as opening and drainage of the sigmoid sinus and packing with the temporal muscle or the fascia lata.1,4-6 Notwithstanding, other authors advocate a more conservative approach, identifying and clearing the granulation tissue and the mastoid osteitis, without needle punching or opening the sigmoid sinus.^7^

The thrombus surgical approach bears its own risks such as septic embolism, dura matter violation with extension to the subarachnoidal space, and it makes it difficult to have spontaneous recanalization in some cases. Therefore, and because of a greater efficacy of antibiotics, there is a current trend among American authors to be more conservative.^7^

Regarding the use of thrombolitic agents, there is much controversy. Some authors advocate their use routinely^1^,^6^,^8^,^9^ and others do not believe they would be efficient.^5^,^7^,^10^ Even among the authors who advocate their routine use, there is an understanding that the ideal anticoagulation therapeutic level is not reached for most of the patients treated with heparin.1 Now, the sigmoid sinus recanalization may occur even without the use of oral anticoagulants.^7^

We carried out a thrombolitic treatment in the three first cases; however, there was a lengthening in hospital stay and the anticoagulation therapy level control was irregular, and since we were not seeing any recanalization of the sigmoid sinus in these patients, we stopped doing it in our most recent cases.

Some authors suggest that only in those situations of neurological decline, including changes in conscience level, is when one must attempt approaches such as thrombolytic agents or surgical thrombectomy^10^; and the internal jugular vein ligature should be reserved only for those cases of septic embolism, regardless of antibiotic treatment.^1^

## CONCLUSIONS

In our series, the SSST diagnoses have been carried out in patients with chronic otitis media always associated with other complications, especially mastoiditis and intracranial abscess. We believe that the sigmoid sinus thrombosis is being underdiagnosed. Although it is a severe problem, clinical prognosis has been favorable with clinical and surgical treatments. Mastoidectomy together with large spectrum antibiotics has been the treatment of choice. We did not see any benefit in those patients who received anticoagulants, when compared to those who did not receive it, despite our small sample. The sigmoid sinus recanalalization has not occurred in most of the cases, even in those patients who underwent thrombolitic treatment.
